# Reporting Quality of the Abstracts for Randomized Controlled Trials in Pediatric Dentistry

**DOI:** 10.1055/s-0043-1770912

**Published:** 2023-08-08

**Authors:** Vellore Kannan Gopinath, Raghavendra M. Shetty, Apathsakayan Renugalakshmi, Lalli Dharmarajan, Ponnudurai Samuel Gnana Prakash, Jayakumar Jayaraman

**Affiliations:** 1 University of Sharjah, College of Dental Medicine, Department of Preventive and Restorative Dentistry, Sharjah, United Arab Emirates; 2 Department of Clinical Sciences, College of Dentistry, Ajman University, United Arab Emirates; 3 Center of Medical and Bio-allied Health Sciences Research, Ajman University, United Arab Emirates; 4 Adjunct Faculty, Department of Pediatric and Preventive Dentistry, Sharad Pawar Dental College and Hospital, Datta Meghe Institute of Higher Education and Research (Declared as Deemed-to-be University), Wardha, Maharashtra, India; 5 Department of Preventive Dental Sciences, Division of Pedodontics, College of Dentistry, Jazan University, Jazan, Saudi Arabia; 6 Department of Pediatric and Preventive Dentistry, Saveetha Institute of Medical and Technical Sciences, Saveetha University, Chennai, India; 7 Department of Periodontics, S.R.M Dental College, Ramapuram, Chennai 600089, Tamil Nadu, India; 8 Department of Pediatric Dentistry, Virginia Commonwealth University School of Dentistry, Richmond 23298, Virginia, United States

**Keywords:** abstract, Pediatric Dentistry, reporting quality, randomized controlled trials

## Abstract

**Objectives** The purpose of this study is to systematically appraise the reporting quality of abstracts for randomized controlled trials (RCT) published in pediatric dentistry using Consolidated Standards of Reporting Trials (CONSORT) for abstracts and to analyze the relationship between the characteristics of the RCT to the quality of abstracts.

**Materials and Methods** RCTs published in Pediatric Dentistry were retrieved from the PubMed database from 2016 to 2021. The quality of abstracts was appraised using CONSORT for abstracts checklist by two independent reviewers.

**Statistical Analysis** In descriptive statistics, frequency and percentage analysis were used for categorical variables, whereas mean and standard deviation were used for continuous variables. To find the significant difference between the bivariate samples in independent groups, Mann–Whitney U test was employed. Multivariate analysis was performed using Kruskal–Wallis test and Mann–Whitney U tests. Probability value of *p* -value less than 0.05 was considered as statistically significant.

**Results** Two hundred abstracts were included in the study. All the abstracts adequately reported the “objective” item, whereas only 2 and 4% of abstracts adequately addressed “randomization” and “harms” items, respectively. A significant relationship was observed between the continent of first author/corresponding author, number of authors, impact factor, adherence to CONSORT guidelines, word count, focus of study, and *a* *priori* protocol registration to the quality of abstracts ( *p* < 0.05).

**Conclusion** The abstracts of the RCT included in the study did not adequately follow the CONSORT for abstract guidelines. Adherence to the reporting guidelines would improve the overall reporting quality of abstracts of RCT published in Pediatric Dentistry. The overall mean score of the abstracts was 6.80 out of 15 indicating that the abstracts did not adequately follow the CONSORT for abstract reporting guidelines.

## Introduction

Randomized controlled trials (RCT) are designed to evaluate the most effective intervention for a disease. 1 2 The results of the RCTs are the most accurate, reliable, and positioned at the highest level, due to their unbiased study design. 1 Abstracts are meant to provide an accurate and brief overview of the work, allowing the clinicians and researchers to gather overall information of the research with no need to read the entire manuscript. 3 The abstract of RCTs provides the readers an insight to enable the selection and evaluation of the research. 3 4 They are of vital importance as the full-text article published in a journal, may not be freely available for online access due to the publisher's terms and conditions, requirements for payment or subscription, etc. Hence, researchers, clinicians, academicians, reviewers, and stakeholders usually depend on the information provided in abstracts. 2 Therefore, the abstract is usually published in the English language to create an impact by reaching a larger audience. 5

Well-reported abstracts of RCTs are important so that the researchers and clinicians can acquire adequate information about the safety, adequacy, and transparency of the clinical trial just by reading an abstract. Poorly designed and reported RCTs are likely to result in inaccurate conclusions and subsequently affect the clinical decision-making process. 6 7 Substandard reporting of the RCT abstracts can result in misinterpretation and poor patient outcomes. 8 Hopewell et al 2 recognized the importance of well-reported abstracts and developed a list of essential items, which authors should consider when reporting the conference or journal abstract of RCTs termed as Consolidated Standards of Reporting Trials for Abstracts. CONSORT for abstract checklist has 17 items that include Title, Authors, Trial Design, Participants, Interventions, Objective, Outcome, Randomization, Blinding, Numbers randomized, Recruitment, Numbers analyzed, Outcome, Harms, Conclusions, Trial registration and Funding. The “Author information” and “Trial status” items are mainly needed for conference abstracts. 9

Recently, Jayaraman 10 described the relevance and importance of CONSORT reporting guidelines for randomized clinical trials in Pediatric Dentistry (PD). A large proportion of RCTs in Pediatric Dentistry show a lack of transparency and reproducibility. 11 In addition to guidelines for reporting different study designs, the RAPID (Reporting stAndards for research in Pediatric Dentistry) statement was recently developed for specific themes in PD and drafted a checklist of items under each theme. 12 Despite over 600 medical and dental journals having already adopted the CONSORT for abstract guidelines, the quality of reporting the RCT abstract remains inadequate. 13 14 15 This reiterates the need to adhere to the reporting guidelines for both medical and dental journals. 16 17

Seehra et al 13 evaluated 228 RCT abstracts published in dental specialty using the modified checklist of CONSORT for abstract and reported that the overall reporting quality score was suboptimal (62.5%). They found that the title, participants, outcomes, numbers randomized, random number generation, and estimation of effect size items were inadequately reported. However, allocation concealment, numbers analyzed, confidence intervals, blinding, harms, trial registration, and funding were rarely described. Alharbi and Almuzian 14 assessed 224 RCT abstracts published from 2012 to 2017 in four major orthodontics journals and reported that items like title, trial registration, numbers analyzed, and funding were moderately adequate and items like blinding and harms were poorly reported. Based on 434 RCT abstracts published in periodontology and implant dentistry journals from 2016 to 2021, the authors indicated an overall suboptimal reporting quality with poor reporting of participant allocation (2%), blinding (3%), and trial registration (4%) items indicating scope for improvements. 15 Similarly, the RCT abstracts in endodontics were assessed by Fang et al, 18 which suggested that the reporting quality must be improved. To the best of the authors' knowledge, there are no studies reported in the literature appraising the standards of abstract of RCTs in PD. Hence, the aim of this study was twofolds:

1. To appraise the reporting quality of RCTs published in PD using CONSORT for abstract checklist, and
2. To analyze the relationship between the continent of first author/corresponding author, year, number of authors, impact factor, adherence to CONSORT for abstract checklist, word count, PD specialty versus Non-Pediatric Dentistry journals, focus of study, and *a* *priori* protocol registration to the reporting quality of RCTs.

## Materials and Methods

### Randomized Controlled Trial Selection Process

RCTs published in PD were retrieved from the PubMed database from January 2016 to August, 2021 using the following search strategy: (((((((((“'randomized controlled trial”) OR (“randomized controlled trial”)) OR (“clinical trial”)) OR (“randomized clinical trial”)) OR (“randomized clinical trial”)) OR (“controlled clinical trial”)) AND (“Pediatric Dentistry”)) OR (“Pediatric dentistry”)) OR (“Children Dentistry”)) OR (“Pedodontics”). The publication details of the randomized clinical trials searched in the databases were exported to an Excel worksheet. A sequence of random numbers between 0 and 1 with four decimal places was generated and assigned to the list of publications. This was sorted in increasing order of the random numbers leading to a successful randomized list of publications. The first 200 publications were considered for evaluation through abstract reading. If any publication did not meet the criteria, it was removed from the list and another sequenced publication was included. A third reviewer (JJ) arbitrated if any disputes occurred in the selection between first and second reviewers (VKG, RMS).

### Selection Criteria

Abstracts of randomized controlled trials in PD where the child's age was less than 17 years were included. No restriction was applied to the journal and language of publication. Case reports/series, retrospective cohort studies, observational, controlled clinical trials, methodology studies (study that dealt with design and conduct of RCT), animal studies, laboratory-based studies, reviews, and conference abstracts were excluded.

### Data Extraction Process

A data extraction sheet was created that included name of the first author, the continent of first author/corresponding author, year published, number of authors, name of the journal and whether the journal has an impact factor based on *Journal Citation Reports* , journal adhered to CONSORT for abstract guidelines and abstract word count. Data were extracted independently by two reviewers and any disagreement between them was resolved by a third reviewer.

### Preliminary Training of Examiners

To standardize the examiners, a training session was conducted, in which examiners discussed about CONSORT for abstract checklist. Following the training session, 10% of the identified abstracts were selected randomly and scored by two examiners (VKG, RMS) independently using the CONSORT for abstract checklist. Any disagreement between them was resolved by discussion or with the help of a third reviewer (JJ). This process served as a pilot for reviewing the abstract using the CONSORT for abstract checklist and allowed concerns with the methodology to be resolved.

### Appraising the Quality of Abstracts

In this study, the quality of abstracts was appraised using CONSORT for Reporting Randomized Controlled Trials in Journal and Conference Abstracts. 2 In general, CONSORT for abstracts has 17 items. However, two items (author information and trial status) were mainly needed for conference abstracts. 9 Hence, those two items were excluded, and the abstracts were appraised using 15 items. 9 A score of “1” was awarded to each item when the abstract satisfied the relevant criteria; if the item was not reported, a score of “0” was awarded. Two reviewers were independently involved in appraising the abstracts and any disagreement between them was resolved by a third reviewer. Intrarater and inter-rater reliability were calculated using Kappa statistics. Intrarater examiner reliability was conducted by evaluating the scores of each examiner for abstracts at the beginning of the study and again after 3 weeks.

### Statistical Analysis

The collected data were analyzed with IBM SPSS Statistics for Windows, Version 23.0. (IBM Corp, Armonk, New York, United States). In descriptive statistics, frequency and percentage analysis were used for categorical variables, whereas mean and standard deviation were used for continuous variables. To find the significant difference between the bivariate samples in independent groups, the Mann–Whitney U test was used. For the multivariate analysis, Kruskal–Wallis test followed by Mann–Whitney U tests was employed. In all the above statistical tools, the probability value of *p* -value less than 0.05 was considered as statistically significant.

## Results

### Literature Search

**Fig. 1:**
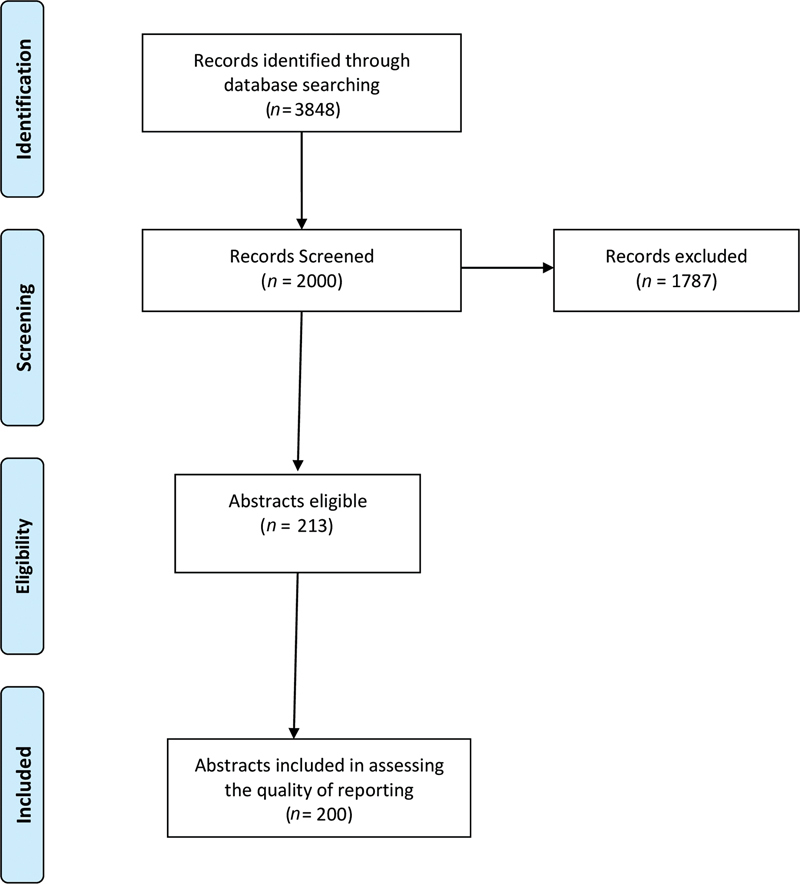
Flowchart indicating the selected randomized controlled trial abstracts for the assessment of quality of reporting.

The process of selecting the abstract for this study is described in Fig. 1 . The initial search in PubMed database resulted 3,848 articles for title/abstract selection. Based on the selection criteria first, 200 abstracts were included for this study. Since a limited number of studies represented Oceania and Africa, three studies representing these continents were excluded from statistical analysis. 19 20 21 Out of 200 studies, only 197 studies were used to assess the relationship comparing first author and corresponding authors continents to reporting quality of RCT abstracts ( Table 1 ).

**Table 1:** Relationship between characteristics of the abstracts and reporting quality

Characteristics	Number (percentage)	Mean ± SD	*p* -Value
**Year of publication (** ***n*** ** = 200)**			
2016	11 (5.5)	6.50 ± 1.6	0.108
2017	36 (18)	6.30 ± 1.3	
2018	40 (20)	6.90 ± 1.3	
2019	29 (14.5)	6.50 ± 1.4	
2020	54 (27)	7.00 ± 1.9	
2021	30 (15)	7.40 ± 2.2	
**Continent of first author (** ***n*** ** = 197) a**			
Asia	87 (44.2)	6.06 ± 1.16	0.0005 **
Europe	28 (14.2)	8.50 ± 2.17	
Middle East	55 (27.9)	6.85 ± 1.28	
North America & Canada	10 (5.1)	7.30 ± 1.34	
South America	17 (8.6)	6.94 ± 1.48	
**Continent of corresponding author (** ***n*** ** = 197) a**			
Asia	84 (42.6)	6.06 ± 1.18	0.0005 **
Europe	29 (14.7)	8.31 ± 2.14	
Middle East	54 (27.4)	6.85 ± 1.29	
North America & Canada	14 (7.1)	7.50 ± 1.74	
South America	16 (8.1)	6.75 ± 1.29	
**Number of authors (** ***n*** ** = 200)**			
1-3	56 (28)	6.60 ± 1.4	0.033 *
4-5	74 (37)	6.60 ± 1.5	
≥ 6	70 (35)	7.20 ± 2	
**Journal with and without impact factor (** ***n*** ** = 200)**			
Yes	94 (47)	7.50 ± 1.9	0.0005 **
No	106 (53)	6.20 ± 1.2	
**Journal adherent to CONSORT for abstract guideline (** ***n*** ** = 200)**			
Yes	129 (64.5)	7.23 ± 1.80	0.0005 **
No	71 (35.5)	6.03 ± 1.01	
**Word count (** ***n*** ** = 200)**			
≤200	46 (23)	6.10 ± 1.2	0.001 *
201–250	75 (37.5)	6.60 ± 1.4	
251–300	49 (24.5)	7.00 ± 1.4	
> 300	30 (15)	8.10 ± 2.5	
**Pediatric Dentistry speciality journal (*n*** **=** **200)**			
Yes	75 (37.5)	6.60 ± 1.4	0.371
No	125 (62.5)	6.90 ± 1.8	
**Focus of the study (** ***n*** ** = 200)**			
Behavior management	52 (26)	6.71 ± 1.09	0.009 *
Preventive	55 (27.5)	6.64 ± 1.56	
Restorative	34 (17)	7.44 ± 2.34	
Pediatric endodontics	46 (23)	6.26 ± 1.25	
Orthodontics	9 (4.5)	8.56 ± 2.35	
Others	4 (2)	7.25 ± 1.71	
**Protocol registered in a clinical trial registry (** ***n*** ** = 200)**			
Yes	30 (15)	9.00 ± 1.9	0.0005 **
No	170 (85)	6.40 ± 1.3	

Abbreviations: CONSORT, Consolidated Standards of Reporting Trials; SD, standard deviation.

* Significant, **Highly significant.

a Since limited number of studies represented Oceania ( *n* = 2) and Africa ( *n* = 1), they were excluded for analysis.

### Interexaminer Reliability

The interexaminer reliability among the reviewers was assessed using Kappa analysis and the score was 0.92 ( *p* < 0.001) equating to “almost perfect” agreement.

### Characteristics of Included Abstracts

The mean CONSORT scores of 200 abstracts of randomized controlled clinical trials in PD were matched with year of publication (2016–2021), continent of first/corresponding authors, number of authors (1-3,4-5, ≥6), journal impact factor, journal adherent to CONSORT guidelines, word count (≥ 200,201-250,251-300, > 300), PD specialty journal, focus of study, and clinical trial registration ( Table 1 ).

### Reporting Quality of Abstracts

The percentage of adequately reported individual items and subitems are presented in Table 2 . The mean overall score of 200 abstracts was 6.805 ± 1.667. Only 2% of abstracts adequately addressed the “randomisation” and 4% addressed “harms” items, respectively. All the abstracts adequately reported the “objective” item.

**Table 2:** Percentage of adequately reported items

Items	Criteria	CONSORT score ( *N* = 200), *n* (%)
Title	Identification of the study as randomized	123 (61.5)
Trial design	Description of the trial design (e.g., parallel, cluster, non-inferiority)	68 (34)
**Method**		
Participants	Eligibility criteria for participants and the settings where the data were collected	32 (16)
	Eligibility criteria for participants	184 (92)
	Setting where the data were collected	32 (16)
Intervention	Interventions intended for each group	195 (97.5)
	Experimental group	196 (98)
	Comparison group	195 (97.5)
Objective	Specific objective or hypothesis	200 (100)
Outcome	Clearly defined primary outcome for this report	191 (95.5)
	Primary outcome	196 (98)
	Outcome—time frame	191 (95.5)
Randomization	How participants were allocated to interventions	4 (2)
	Random assignment	177 (88.5)
	Method of randomization	12 (6)
	Concealment	5 (2.5)
Blinding	Whether or not participants, care givers, and those assessing the outcome were blinded to group assignment	22 (11)
	Generic description only (e.g., single-blind, double-blind)	35 (17.5)
**Results**		
Numbers randomized	Number of participants randomized to each group	179 (89.5)
Numbers analyzed	Number of participants analyzed in each group	80 (40)
Outcome	For the primary outcome, a result for each group and estimated effect size and its precision	16 (8)
	Primary outcome results for each group	199 (99.5)
	Estimated effect size	18 (9)
	Precision of the estimation (e.g., 95% CI)	25 (12.5)
Harms	Important adverse events or side effects	8 (4)
Conclusion	General interpretation of the results	199 (99.5)
Trial Registration	Registration number and name of trial register	23 (11.5)
	Trial registration number	31 (15.5)
	Trial registration name	23 (11.5)
Funding	Source of funding	21 (10.5)

Abbreviations: CI, confidence interval; CONSORT, Consolidated Standards of Reporting Trials.

## Relationship between Characteristics of the Trials and the Reporting Quality

### Year of Publication

No significant difference was observed between year of publication and the reporting quality of RCT abstracts. The RCTs published in 2021 (7.4) and 2020 (7.0) reported higher mean CONSORT-A scores compared to 2016 (6.5), 2017 (6.3) 2018 (6.9), and 2019 (6.5) ( Table 1 ).

### Continent of the First Author

Among 197 abstracts included, a significant difference was observed among North America and Canada, South America, Europe, Asia, and Middle East continents ( *p* =0.0005; Table 1 ). The reporting quality of RCT abstract published from Europe (8.50) was with high mean CONSORT-A scores compared to Asia (6.06), Middle East (6.85), and South America (6.94). Among continents, Asia scored the least mean score value compared to Europe, Middle East, North America and Canada, and South America ( *p* < 0.05). However, no significant difference was found while comparing North America and Canada to South America; Middle East compared to North and South America; Europe to North America and Canada ( *p* > 0.05).

### Continent of the Corresponding Author

Based on 197 abstracts, a significant difference was observed among North America and Canada, South America, Europe, Asia and Middle East continents ( *p* =0.0005; Table 1 ). An overall high mean abstract CONSORT-A score was seen in clinical trials published in Europe that was significantly high compared to Asia, Middle East, and South America ( *p* < 0.05). Although Asia scored low compared to Europe, Middle East, North America and Canada ( *p* < 0.05), no significant difference in the mean scores was observed between Asia and South America ( *p* > 0.05). The difference was not statistically significant between Europe, North America and Canada; Middle East and North America and Canada; Middle East and South America; North America and Canada and South America ( *p* > 0.05).

### Number of Authors

A significant difference ( *p* = 0.033) was observed among the number of authors ( Table 1 ). Trial abstracts mean CONSORT sores with more than or equal to 6 authors were high compared to 1 to 3 ( *p* = 0.041) and 4 to 5 authors ( *p* = 0.016). No statistical difference in mean sores was observed between 1 to 3 and 4 to 5 authors.

### Impact Factor

A significant difference was observed between trial abstract published in journals with impact factor compared to no impact factor ( *p* = 0.0005; Table 1 ). The mean abstract CONSORT- A scores in journals with impact factor was higher.

### Journal adherence to CONSORT Guidelines

A significant difference was observed between journals adhering and not adhering to consort guidelines ( *p* = 0.0005; Table 1 ). Trial abstracts published in journals that adhered to guidelines had a better score.

### Word Count

A significant difference was observed between word count ( *p* = 0.001) ( Table 1 ). The mean CONSORT scores in abstract with word count less than or equal to 200 was significantly lower when compared to abstract with 251 to 300 and more than 300 words, also word count of 201 to 250 was lower compared to more than 300 ( *p* < 0.05) words. However, no significant difference was seen between word count of less than or equal to 200 to 201 to 250; 201 to 250 to 251 to 300; 251 to 300 to more than 300 ( *p* > 0.05).

### Pediatric Dentistry Specialty versus Non-Pediatric Dentistry Specialty Journals

No significant difference was observed ( *p* = 0.371) in the journal category. The mean CONSORT scores of trial abstracts published in non-Pediatric Specialty journals (6.9) were slightly higher compared to PD journals (6.6) but not statistically significant ( *p* > 0.05; Table 1 ).

### Focus of the Study

A significant difference was observed between the focus of the study and mean CONSORT scores. Trial abstracts focusing on orthodontics (8.56) and restorative dentistry (7.44) had higher scores ( Table 1 ). Similarly, studies in orthodontics had higher mean scores compared to pediatric endodontics ( *p* = 0.003); restorative compared to pediatric endodontics ( *p* = 0.012); orthodontics compared to preventive ( *p* = 0.012).

### *A priori* Protocol Registration

A significant difference was observed between studies where protocol registration was done compared to no registration ( *p* = 0.0005). In the present sample, registered trials had a higher mean abstract score compared to nonregistered trials ( Table 1 ).

## Discussion

To our understanding, this is the first study to analyze the reporting quality of abstracts of RCTS in PD using CONSORT for abstracts. Considering the importance of abstracts, particularly pertaining to RCTs, several studies have evaluated the reporting of RCT abstracts quality in medical journals with an overall adherence rate of 67% among general medicine journals 22 and 53% for anesthesia journals. 23 This has also been reported in dentistry including orthodontics 24 and periodontics 9 25 and combined specialties. 13 Consistent with the above studies, the reporting quality of abstracts of RCTs in PD was suboptimal and requires substantial improvement in several areas.

The CONSORT for abstract checklist comprises of 17 items in the domains focusing on the title, authors, trial design, methods, results, conclusions, trial registration, and funding. 2 The domain on authors and trial status is only meant for conference abstracts so we excluded these domains. Hence, we included only 15 items of the CONSORT for abstract checklist in this study. 10 In our study, 2 and 4% of abstracts adequately addressed “randomization” and “harms” items. It is important to report “randomization” in the abstract as this is considered as an important part of a trial that determines how participants were allocated toward the intervention. This is usually done through sequence generation. Another aspect of randomization is allocation concealment that primarily helps to avoid selection bias of the participants. Similarly, “harms” item reports the important adverse events or side effects. This enables to make balanced decisions considering both benefits and harms of an intervention. The authors must explicitly report any adverse events that was observed in the trial. 2

In PD, two studies have evaluated the reporting quality of randomized controlled trials published between 1985 and 2006, followed by those published between 2011 and 2012. Both studies found that reporting of clinical trials was poor, and they have not improved since the publication of CONSORT guideline. 26 27 Considering these inadequacies, a detailed report was published in 2020 on the importance of adhering to CONSORT guidelines that provided guidance to the authors on reporting randomized trials in PD. 10 Like this study, in 2019, a study was published that analyzed the reporting quality of abstracts of systematic reviews and meta-analysis in PD journals based on the 12-item PRISMA-A (Preferred Reporting Items for Systematic Review and Meta-Analysis – Abstract) checklist and found that the abstracts were of moderate quality. 28 All the studies on abstracts across different disciplines and study designs clearly indicate that the reporting quality has not improved since the implementation of the CONSORT for abstract checklist.

In our study, the number of words in the abstracts had a significant effect on the outcome scores, with a mean score of 6.1 and 8.1 for abstracts lesser than 200 words and more than 300 words, respectively. Most of the specialty journals in PD have word restrictions for abstract; for example, the number of words for summary or abstract for International Journal of Pediatric Dentistry is 200 (IJPD), and 200 to 250 words for PD journal. For European Archives of Pediatric Dentistry, the required word count is between 150 and 250. It is to be noted that all the above journals have specific recommendations to follow CONSORT in the “Submission Guidelines” when submitting the article to the journals. We recommend that the journals should make it mandatory for the authors to submit a CONSORT checklist with appropriate reference to the pages in the manuscript to ensure that all the guidelines have been reported in the manuscript, including the abstract. In addition, we recommend that the journals should provide more allocation for words to be able to have all the reporting items of the CONSORT checklist included in the abstract.

The overall mean score of the CONSORT in our study was 6.80, 1.667 (mean, standard deviation). This was consistent with a study conducted in psychiatry specialty that reported a mean score of 6.90 for pre-CONSORT publications 29 but better than general dental journals with a mean score of 4.53. 30 It is to be noted all the trial abstracts assessed in our study were post-CONSORT and published between 2016 and 2021. The mean score of the studies published in 2016 was 6.5 and did not differ significantly compared to those published in 2021 with a mean score of 7.4. The way the outcomes were analyzed varied across different studies; for example, few reported the CONSORT for abstract adherence score in mean and standard deviation, 29 while others reported in median and interquartile range 9 and in percentages. 24 25 Hence, it was not possible to compare the mean scores of our study with other studies that reported the outcomes differently.

It is to be noted that all the studies that evaluated the reporting quality of abstracts of RCT in dentistry restricted their search to only specialty journals. 9 24 We feel this might have excluded valuable data published in nonspecialty journals. Hence, in this study, we included both specialty and nonspecialty journals that published randomized trials in PD. It is interesting that we did not find a significant difference in the outcomes of CONSORT scores between the above journals.

Our study has a few limitations; the sample is representative of articles published in the last 5 years and hence it reflects the characteristics of the articles published recently. Nevertheless, further studies can be conducted to note if there are any wide discrepancies in reporting quality between studies published before and after the release of CONSORT for abstract guidelines. Our study has some strengths. Unlike previous publications, our samples represented RCT published in PD specialty and nonspecialty journals that were retrieved from PubMed database and not limited to high-ranking journals.

Reporting quality of abstracts of systematic reviews and meta-analysis in PD has been already reported 28 and the authors in this study evaluated the reporting quality of abstracts of RCT. Future studies should focus on evaluating the abstracts of other study designs including cross-sectional studies, laboratory studies, and observational studies in Pediatric Dentistry. Despite the release of the CONSORT for abstract statement a decade ago, it is worth mentioning that the reporting quality of the studies published in PD journals is suboptimal and needs to be improved. Unanimous efforts are to be adopted by the reviewers and editors to ensure that the authors comply with the CONSORT requirements. Academicians, researchers, and clinicians need to be proficient in presenting their data in a more standardized and transparent method.

## Conclusion

The abstracts of the RCT included in the study had a mean score of 6.80 out of 15 and did not adequately follow the CONSORT for abstract guidelines. No difference was found in the reporting quality of studies published between non-specialty and PD journals. The reporting quality was higher for journals published by the first and corresponding authors from Europe, and for abstracts that had a word count of over 300 words. Adherence to the reporting guidelines would improve the overall reporting quality of abstracts of RCT published in PD.
